# Dexamethasone reduces autoantibody levels in MRL/lpr mice by inhibiting Tfh cell responses

**DOI:** 10.1111/jcmm.16785

**Published:** 2021-07-28

**Authors:** Chunxiu Shen, Xiaonan Xue, Xiaoyu Zhang, Lihua Wu, Xiangguo Duan, Chunxia Su

**Affiliations:** ^1^ School of Basic Medical Sciences Ningxia Medical University Yinchuan China; ^2^ Department of Nephrology General Hospital of Ningxia Medical University Yinchuan China; ^3^ Department of Laboratory Surgery General Hospital of Ningxia Medical University Yinchuan China; ^4^ College of Clinical Medicine Ningxia Medical University Yinchuan China

**Keywords:** dexamethasone (Dex), MRL/lpr mice, SLE, T follicular helper (Tfh) cells

## Abstract

Previous studies have shown that dexamethasone (Dex) reduces the levels of anti‐nuclear (ANA) and anti‐dsDNA antibodies in MRL/lpr mice (a mouse model of SLE). However, the effect of Dex on T follicular helper (Tfh) cells is less documented. Here, using the MRL/lpr mouse model, we investigated the influence of Dex on Tfh cells and potential underlying mechanisms. The data showed that the proportion of Tfh cells, identified as CD4^+^CXCR5^+^ICOS^+^, CD4^+^CXCR5^+^PD‐1^+^ or CD4^+^BCL‐6^+^ cells, markedly decreased after treatment with the Dex, in both Balb/c mice and MRL/lpr mice. Dex significantly inhibited IL‐21 expression at both the mRNA and the protein levels. Dex also significantly reduced the proportion of germinal centre B cells and decreased serum IgG, IgG2a/b and IgA levels. Moreover, a positive correlation between the proportion of Tfh cells (CD4^+^CXCR5^+^ICOS^+^, CD4^+^CXCR5^+^PD‐1^+^ or CD4^+^BCL‐6^+^) and autoantibodies was observed. Dex significantly increased the *Prdm1* and *Stat5b* mRNA expression and decreased the *Bcl‐6* and *c‐Maf* mRNA expression of CD4^+^T cells. In brief, Dex inhibited the Tfh development, which relies on many other transcription factors in addition to *Bcl‐6*. Our data indicate that Dex can be used as a Tfh cell inhibitor in SLE.

## 1 INTRODUCTION

Systemic lupus erythematosus (SLE) is a chronic autoimmune disease characterized by abundant production of autoantibodies, particularly anti‐nuclear (ANA) and anti‐dsDNA antibodies.^1^, ^2^, ^3^, ^4^ Recently, the role of T follicular helper (Tfh) in antibody production has attracted much attention. Tfh cells are characterized by a high expression of surface molecules, including the chemokine receptor CXCR5, inducible costimulatory molecule (ICOS) and programmed death‐1 (PD‐1).^5^, ^6^, ^7^, ^8^, ^9^ Compared to other CD4^+^T cells, Tfh cells specifically express the transcription factor BCL‐6 and produce the characteristic cytokine IL‐21.^10^, ^11^, ^12^ Tfh cells help to generate germinal centre (GC) reactions where somatic hypermutation and affinity maturation occur, leading to the production of memory B cells and plasma cells.^13^, ^14^, ^15^ Thus, Tfh cells are crucial to GC reactions and high‐affinity antibody production.

In MRL/lpr mice, the abnormally high expression of Tfh‐associated molecules, such as ICOS, PD‐1, BCL‐6 and IL‐21, suggests that Tfh cells may play a crucial role in the pathogenesis of SLE, which is closely connected with autoantibody production and/or lupus‐like symptoms.^15^, ^16^, ^17^, ^18^, ^19^ This observation is reminiscent of the significant correlation between the high proportion of circulating cells displaying a Tfh phenotype, abnormal production of autoantibodies and disease severity in SLE patients.^20^, ^21^ Recent work clearly evidenced an increased frequency of circulating Tfh cells accompanied by higher levels of serum IL‐21 in SLE patients. ^21^, ^22^ Simpson *et al* found that the frequency of circulating Tfh cells is strongly correlated with the number of their counterparts residing in the GC.^23^, ^24^ Therefore, circulating Tfh cells in humans could serve as a biomarker, indicative of the occurrence of such a potential mechanism of GC tolerance disruption. If this hypothesis is verified, it may be an effective therapeutic target for SLE treatment through inhibiting Tfh cell and the GCs response.

At present, glucocorticoids are used as the first‐line drugs for the treatment of SLE. Among them, Dex is often used to treat severe nephritis or other serious organ complications in SLE patients.^25^, ^26^, ^27^ However, it is still unclear how Dex affects Tfh cells in SLE.

Our study aimed to evaluate the efficacy of Dex in modulating Tfh cells during SLE treatment. Our results indicated that Dex down‐regulates Tfh cell responses and decreases the number of Tfh cells by regulating specific transcription factors, which include *Bcl‐6*, *c‐Maf*, *Prdm1* and *Stat5b*. Thus, our research demonstrates that the mechanism whereby Dex inhibits immune responses involves the regulation of Tfh cell transcription factors and the differentiation of B cells and antibody production in the GC.

## 2 MATERIALS AND METHODS

### 2.1 Mice and murine model

Six‐ to eight‐week‐old female MRL/lpr mice and Balb/c mice (from the Shanghai SLAC Laboratory Animal Co., Ltd) were all used in experiment. All 16‐week‐old MRL/lpr and Balb/c mice were randomized into two groups :(1) 1 mg/kg of dexamethasone injection (H42020020; China) in 100 μL normal saline; (2) 100 μL normal saline. The animals received continuously intraperitoneal injections for 4 weeks. Mice were harvested at 20 weeks old.

### 2.2 Cell isolation and culture

Splenocytes were derived from 6‐ to 8‐week‐old female Balb/c mice. Total CD4^+^T cells were selected by CD4^+^T‐cell isolation kit and stimulated with 1 μg/ml anti‐CD3, 1 μg/ml anti‐CD28, 10 ng/mL IL‐21 and 20 ng/mL IL‐2 (all from BD Biosciences) in the presence or absence of the Dex at concentrations of 0.5,1 or 2 μg/mL.^28^ At day 3, we collected the cultured cells and supernatant for the further analysis.

### 2.3 Flow cytometry analysis

The surface antibodies of this experiment were FITC‐conjugated anti‐CD4 antibody, APC‐conjugated anti‐CXCR5 antibody, PE‐conjugated anti‐PD‐1 antibody, and BV421‐conjugated anti‐ICOS antibody, AF488‐conjugated anti‐B220 antibody and AF647‐conjugated anti‐GL‐7 antibody. We also used PerCP‐Cy5.5‐conjugated anti‐BCL‐6 intracellular antibody (all from BD Biosciences). Cell acquisition was performed on a BD FACSCelesta flow cytometer (BD Biosciences).

### 2.4 Using ELISA analysis cytokine and autoantibody titre

Mice serum was collected after intervention. In the light of the manufacturer's guidelines, the total IgG, IgG2a/b, IgA (all from eBioscience), cytokine IL‐21 and autoantibody (from J&l Biological) were analysed using the mouse ELISA Kit.

### 2.5 TaqMan PCR analysis

RNA was isolated using QIAGEN RNeasy Micro Plus Kit (QIAGEN), following manufacturer's guidelines and converted to cDNA by reverse transcription (RT) with random hexamers and Multiscribe RT (Thermo Fisher Scientific). For the expression of mRNA assays, the probes were used: *Il‐21*(Mm00517640_m1), *Bcl‐6*, (Mm00 477633_m1), *c‐Maf*(Mm02581355_s1), *Stat3*(Mm01219775_m1), *Prdm1* (Mm0047 6128_m1), *Stat5b*(Mm00839889_s1); *b‐actin*(Mm02619580_m1) (all from Applied Biosystems). The mRNA expression was assessed by real‐time reverse transcription polymerase chain reaction (RT‐PCR) analysis, according to the manufacturer's instruction.

### 2.6 Histopathology test

Kidney samples were fixed with 4% formaldehyde, dehydration as well as wax immersion, embedded in paraffin and finally cut into 5 mm sections. And then, the sections were washed in deparaffinized and dehydrated via sequential addition of xylene, 100% ethanol, 95% ethanol and distilled water and stained with H&E (all from Sigma). Finally, the slides were evaluated by an experienced pathologist blinded to the treatment protocol. HE staining was used to observe inflammatory cell infiltrates, each with a score of 0‐4 (0, absent; 1, <25%; 2, 25%‐50%; 3, 50%‐75% and 4, >75%).

### 2.7 Immunofluorescence

For the detection of glomerular IgG deposition, 6 μm frozen kidney sections were incubated with FITC‐conjugated goat anti‐mouse IgG (R&D) and then mounted with anti‐fade mounting medium (Beyotime). Semi‐quantitative analysis of glomerular IgG deposition was performed using the following scale (0‐3): 0 = negative staining, 1 = barely visible at high magnification, 2 = moderately visible and 3 = clearly visible.

### 2.8 Statistics

Quantitative data are expressed as means ± SD. Statistical analyses were performed using a Student's t test with SPSS 20.0 software. Correlations were determined by Spearman's ranking. *P*‐value less than 0.05 was considered statistically significant.

## 3 RESULTS

### 3.1 Dex suppresses disease progression in MRL/lpr mice

**FIGURE 1:**
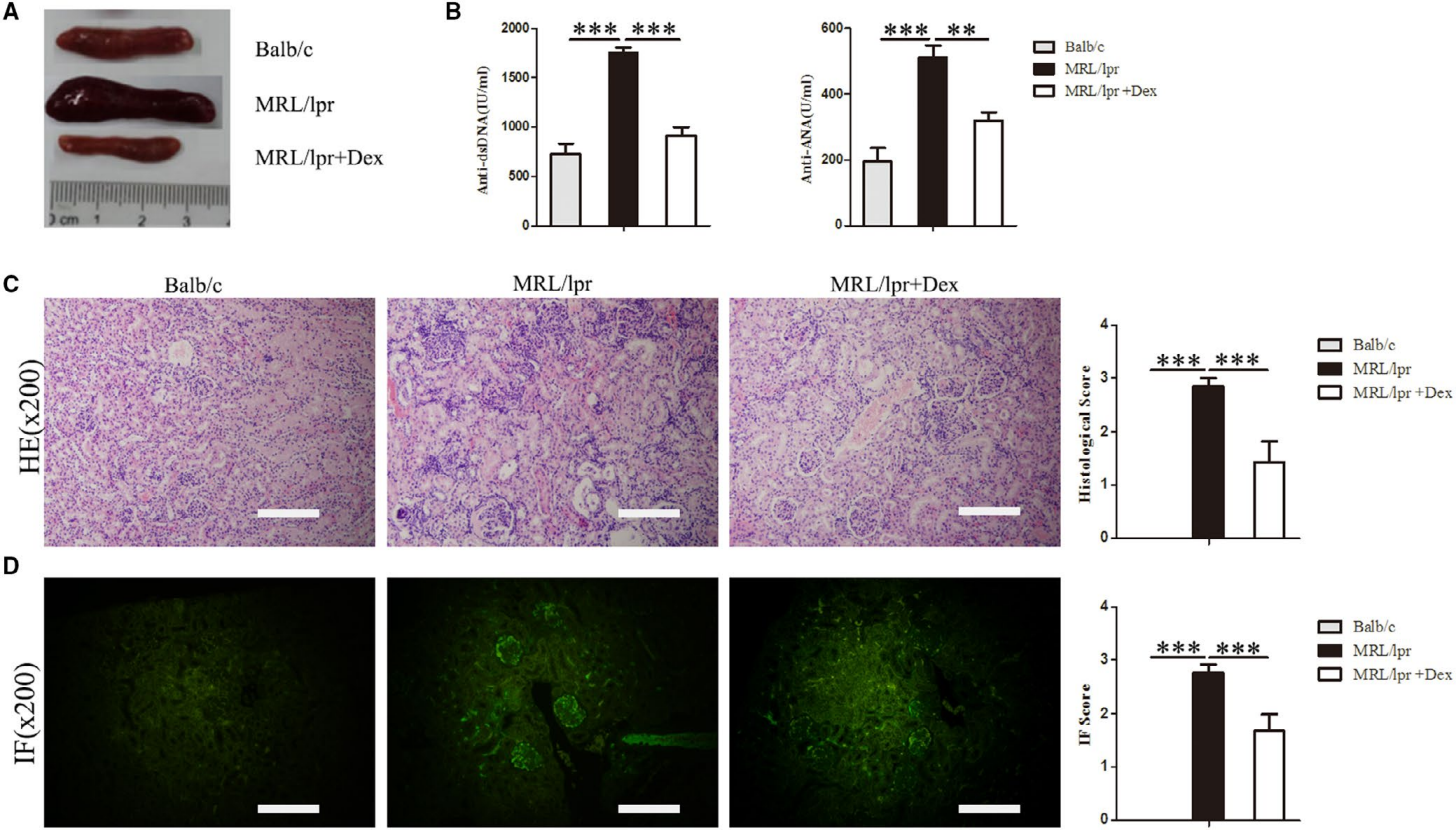
Effects of Dex on the lupus syndromes of MRL/lpr mice. Sixteen‐week‐old female MRL/lpr mice(36±2g) were treated with vehicle (normal saline) or 1 mg/kg of Dex for 4 weeks, age‐matched Balb/c mice as the normal control group. A Splenomegaly in MRL/lpr mice and alleviated after Dex treatment. B The serum levels of anti‐dsDNA antibodies and ANA. C Sections of kidney tissue were stained with H&E and semi‐quantitative analysis of the histological score. D Sections of kidney tissue were stained with Immunofluorescence IgG and semi‐quantitative analysis of glomerular IgG deposition. Original magnification × 200. The scale bar in each image represents 100 μm. Values are the mean and SD of 5 mice per group, **P < .01 and ***P < .001

Previous research found that MRL/lpr mice exhibit splenomegaly, a significant increase of cell proliferation and IgG deposits in kidney glomeruli and high levels of serum ANA and anti‐dsDNA antibodies.^28^, ^29^ Similar to a previous report,^30^ our data showed that the levels of serum ANA and anti‐dsDNA antibodies were significantly reduced after Dex treatment in MRL/lpr mice (Figure 1B). To further evaluate the effects of Dex on disease progression, we determined the size of the spleen, cellular proliferation and IgG deposits in the glomeruli. As shown in Figure 1A, the splenomegaly of MRL/lpr mice was significantly lowered by Dex treatment. Histological analysis also revealed changes between the three groups. Compared with normal mice, MRL/lpr mice showed a significant increase of cell proliferation and IgG deposition in the glomeruli, which were diminished by Dex treatment (Figure 1B,1D).

### 3.2 Dex reduces the frequency of Tfh cells in vitro

**FIGURE 2:**
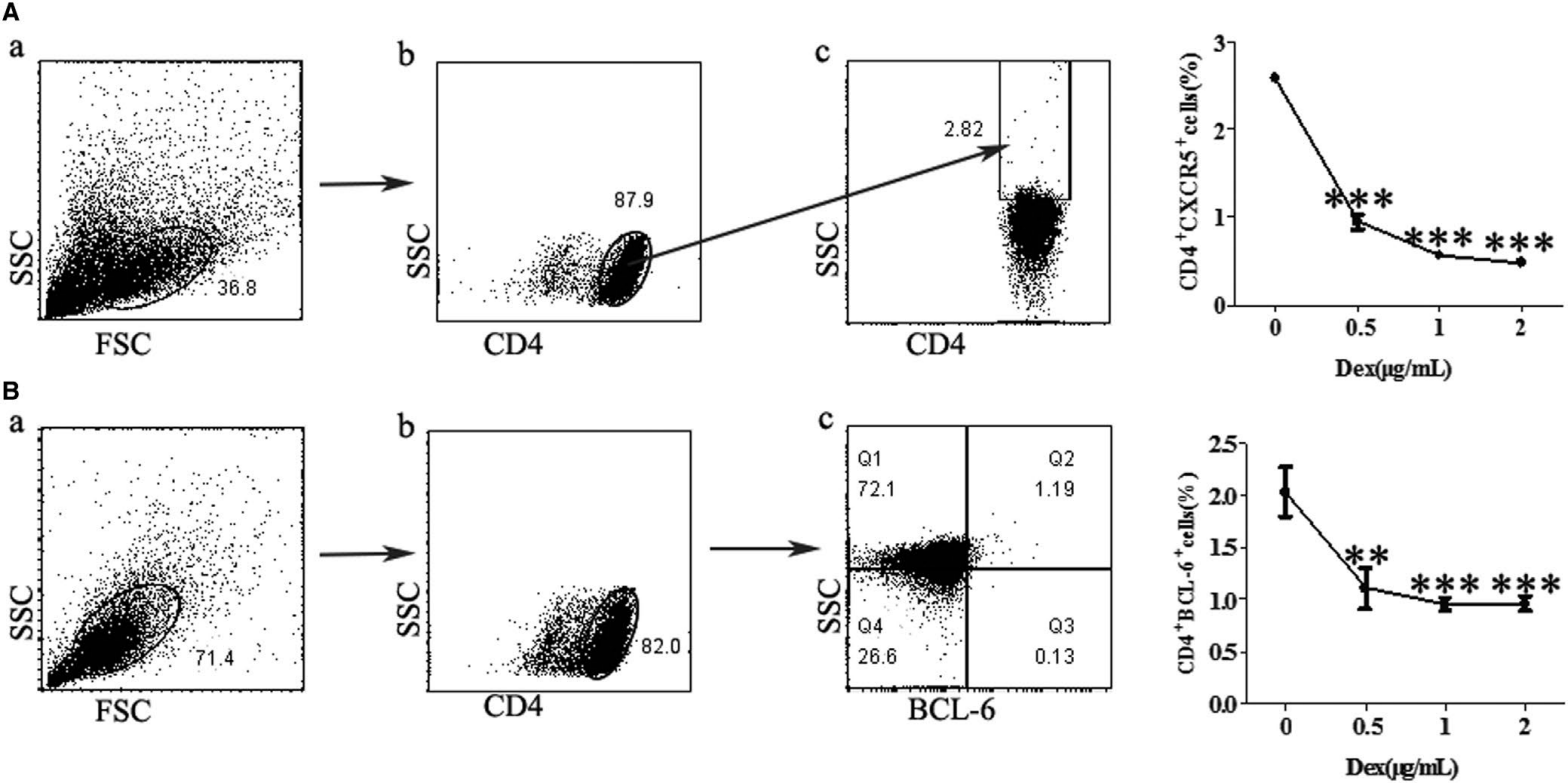
Effects of Dex on Tfh cells in vitro. CD4^+^T cells were stimulated with anti‐CD3, anti‐CD28, IL‐2 and IL‐21 in the presence of different concentrations of Dex for 3 days. (A‐B) Gating strategies used to identify Tfh cells (CXCR5^+^ CD4^+^, or BCL‐6^+^CD4^+^) in vitro. (A) Total CD4^+^ T cells were isolated from Balb/c mice by negative selection using a CD4^+^ T‐cell isolation kit (A, gate a), gated in their totality (A, gate a) or singlets (A, gate b) for the identification of CD4^+^‐positive cells. Finally, CD4^+^ T cells were identified as CXCR5^+^ cells (A, gate c), and the percentage of CD4^+^CXCR5^+^ Tfh cells (B, gate d). (B) Total CD4^+^ T cells were isolated from Balb/c mice by negative selection using a CD4^+^ T‐cell isolation kit (B, gate a), gated in their totality (B, gate a) or singlets (B, gate b) for the identification of CD4^+^‐positive cells. Finally, CD4^+^ T cells were identified as BCL‐6^+^ cells (B, gate c), and the percentage of CD4^+^BCL‐6^+^ Tfh cells (B, gate d). Values are the mean and SD of 3 independent experiments. **P < .01 and ***P < .001

CD4^+^T cells were co‐cultured with anti‐CD3, anti‐CD28, IL‐21and IL‐2 in the presence or absence of Dex for 3 days. After this time, we detected the proportion of Tfh cells by flow cytometry (Figure 2A‐C, 2B‐C). The frequency of Tfh cells, identified as CXCR5^+^CD4^+^cells, decreased significantly and in a dose‐dependent manner upon Dex treatment (Figure 2A‐D). BCL‐6 is critical to Tfh cell differentiation. Comparing the expression of BCL‐6 in cells treated with Dex or vehicle, we found that Dex significantly reduced the expression of BCL‐6 in Tfh cells (Figure 2B‐D).

### 3.3 Dex minimizes the Tfh cell and GC B‐cell responses and antibody production in Balb/c mice

Tfh cells are characterized by increased expression of numerous molecules, including the surface markers CXCR5, PD‐1 and ICOS, and the transcription factor BCL‐6. To investigate whether Dex down‐regulates the expression of these markers on Tfh cells in vivo, we treated 16‐week‐old Balb/c mice with Dex for 4 weeks. We found that the frequency of Tfh cells, identified as CD4^+^CXCR5^+^ICOS^+^, CD4^+^CXCR5^+^PD‐1^+^, or CD4^+^BCL‐6^+^, was significantly reduced after treatment with Dex (Figure S2 2A‐C and Figure S1 1A‐C‐D, B‐B). IL‐21 plays an important role in Tfh cells and GC formation. Thus, we analysed the level of serum IL‐21 and *Il‐21* mRNA expression in CD4^+^T cells from Balb/c mice. We found that IL‐21 expression was down‐regulated at both protein and mRNA levels after treatment with Dex (Figure S2 2E). The development of Tfh cells requires the combined action of numerous transcription factors, such as *Bcl‐6*, *c‐Maf*, *Stat3* and *Prdm1*. We studied the expression of these transcription factors after treatment with Dex. Bcl‐6 expression was down‐regulated. In contrast, the expression of Prdm1 was up‐regulated; there was no detectable effect of the treatment on the expression levels of *Stat3*, *Stat5b* and *c‐Maf* (Figure S2 2F). Overall, our results suggested that Dex could inhibit the differentiation of Tfh cells.

Given that Tfh cells are critical to B‐cell responses and GC formation, we next measured the effects of Dex on B‐cell differentiation. Consistent with its effects on Tfh cells, we observed that treatment with Dex significantly reduced the proportion of B220^+^GL‐7^+^B cells in the spleen (Figure S2 2D and Figure S1 1C‐C). Mirroring the decrease of B‐cell proportion, the levels of serum IgG and IgG2a/b were significantly decreased in Dex‐treated mice. However, Dex had no effect on IgA levels (Figure S2 2G).

### 3.4 Dex suppresses Tfh cells, GC B cells and antibody secretion in MRL/lpr mice

**FIGURE 3:**
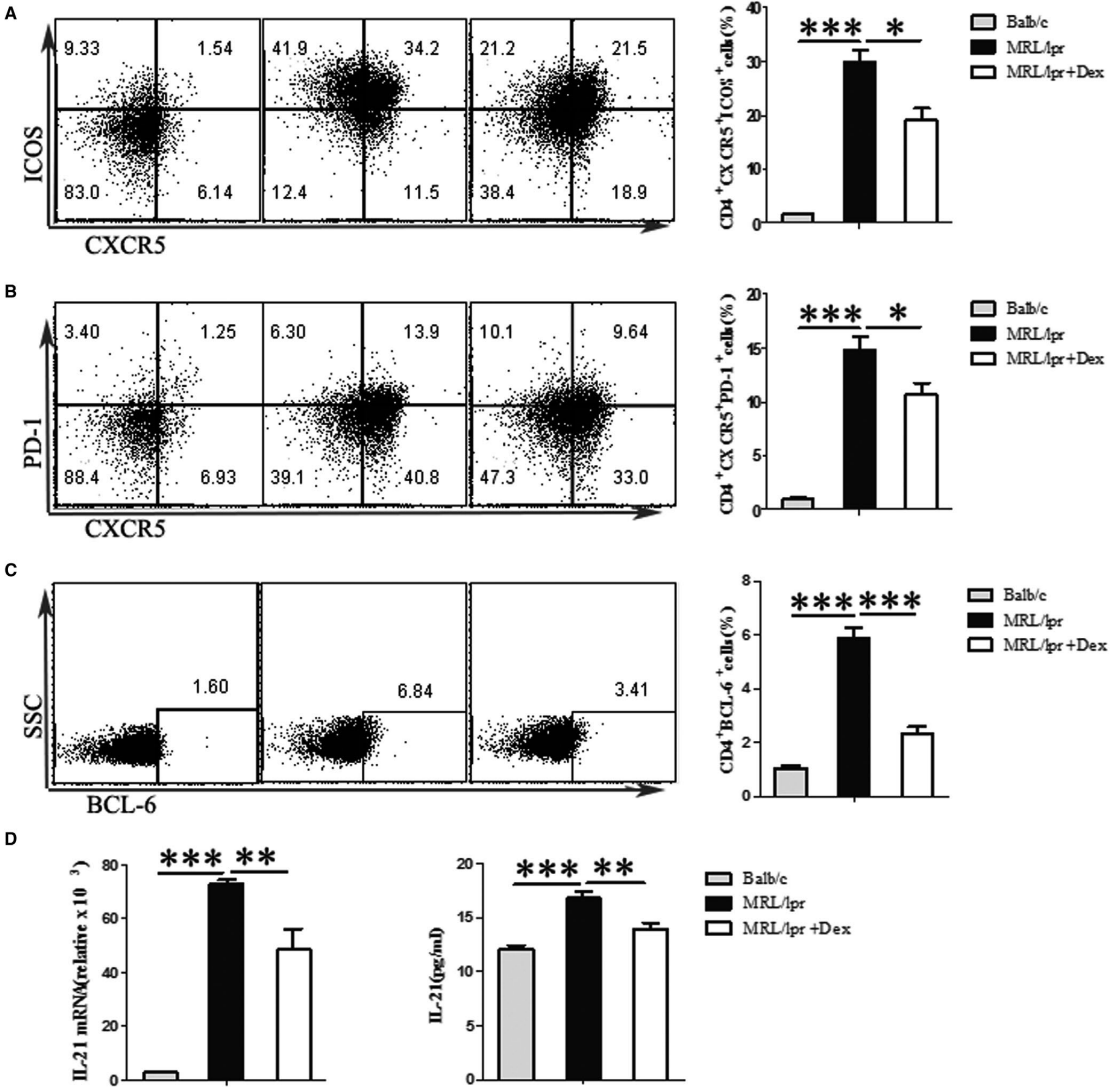
Effects of Dex on Tfh cells in MRL/lpr mice. Sixteen‐week‐old female MRL/lpr mice(36±2g) were treated with vehicle (normal saline) or 1 mg/kg of Dex for 4 weeks, age‐matched Balb/c mice as the normal control group. A Flow cytometric plots (left) of CD4^+^CXCR5^+^ICOS^+^ Tfh cells and a summary graph (right).B Flow cytometric plots (left) of CD4^+^CXCR5^+^PD‐1^+^ Tfh cells and a summary graph (right).C Flow cytometric plots (left) of CD4^+^BCL‐6^+^ Tfh cells and a summary graph (right). D Levels of cytokine in serum of MRL/lpr mice was analysed by ELISA, and mRNA expression of *Il‐21* measured by TaqMan PCR. Values are the mean and SD of 5 mice per group. *P < .05, **P < .01 and ***P < .001

To further investigate the clinical significance of our findings, we tested the effect of Dex on Tfh cell differentiation in MRL/lpr mice. Similar to previous studies that showed that autoimmune MRL/lpr mice exhibited splenomegaly, with the expansion of Tfh cells in the spleen.^22^ Our data indicated MRL/lpr mice had a significantly higher frequency of Tfh cells identified as CD4^+^CXCR5^+^ICOS^+^, CD4^+^CXCR5^+^PD‐1^+^ or CD4^+^BCL‐6^+^ cells as compared to Balb/c mice (Figure 3A‐C and Figure S1 1A‐C‐D, B‐B). Compared to Balb/c mice, the level of IL‐21 also was increased in the serum of MRL/lpr mice, which was correlated with a higher expression of *Il‐21* mRNA (Figure 3D). This observation prompted us to examine whether Dex could influence the expansion of Tfh cells. Our findings demonstrated that the proportion of splenic Tfh cells was significantly decreased in mice treated with Dex (Figure 3A‐C). Furthermore, Dex reduced the protein and mRNA expression of IL‐21 in MRL/lpr mice (Figure 3D).

**FIGURE 4:**
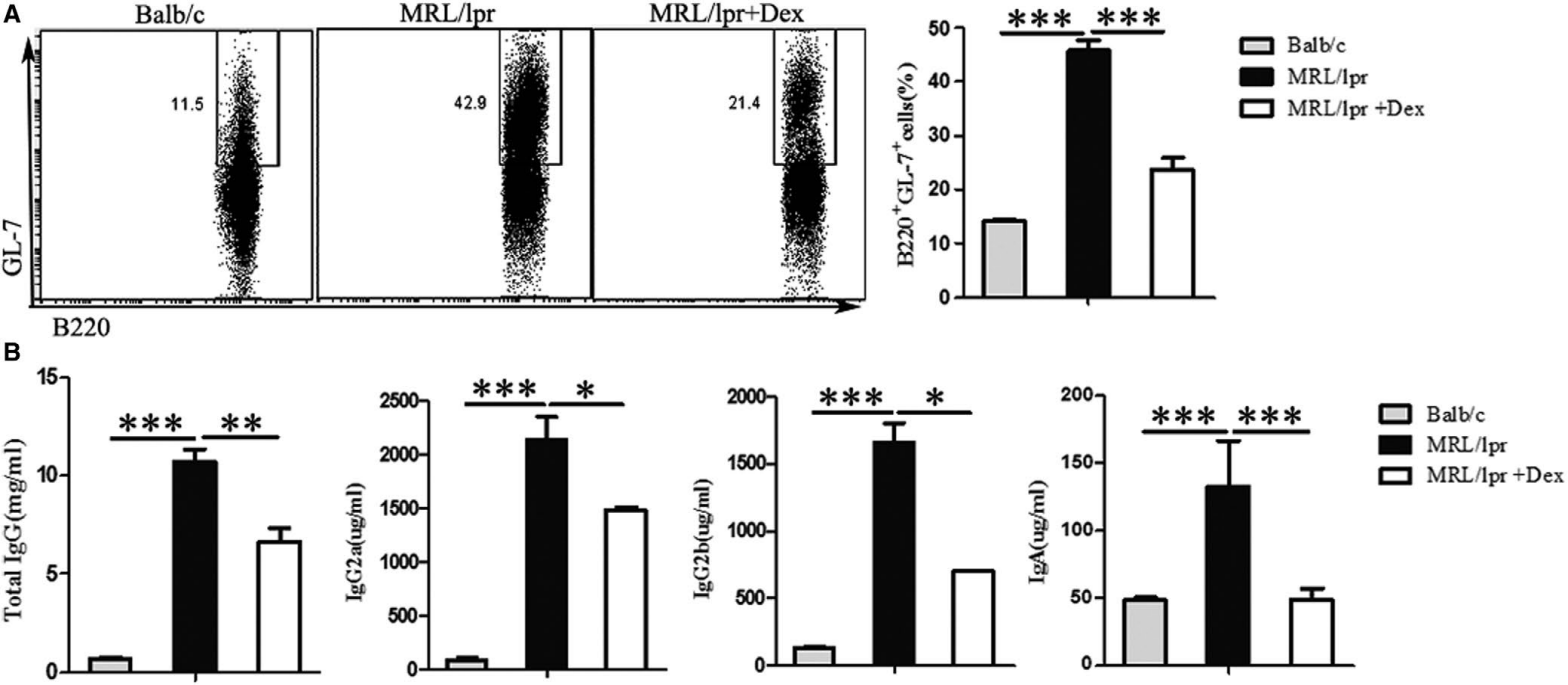
Effects of Dex on GC B‐cell response and antibody secretion in MRL/lpr mice. Sixteen‐week‐old female MRL/lpr mice(36±2g) were treated with vehicle (normal saline) or 1 mg/kg of Dex for 4 weeks, age‐matched Balb/c mice as the normal control group. A Flow cytometric plots (left) of GC B cells in the indicated groups and a summary graph (right). B IL‐21in the serum of the indicated groups as analysed by ELISA, and mRNA expression of *Il‐21* measured by TaqMan PCR. Values are the mean and SD of 5 mice per group, *P < .05, **P < .01 and ***P < .001

An increasing body of evidence shows that the number of GC B cells have a directly correlated with disease activity and B‐cell numbers in MRL/lpr mice.^22^ Compared to Balb/c mice, we found that the frequency of GC B cells was significantly higher in MRL/lpr mice, and this frequency would decrease on treatment with Dex (Figure 4A and Figure S1 1C‐C**)**. Previous research demonstrated a strong positive correlation between the increased number of Tfh cells and the pathogenesis and severity of autoimmune diseases, which are GC‐dependent. Moreover, MRL/lpr mice showed markedly higher levels of IgG, IgG subtypes and IgA than normal mice.^22^ The levels of IgG, IgG2a/b and IgA were markedly decreased in MRL/lpr mice after Dex treatment (Figure 4B).

### 3.5 Dex suppresses disease progression through the inhibition of Tfh cell responses

**FIGURE 5:**
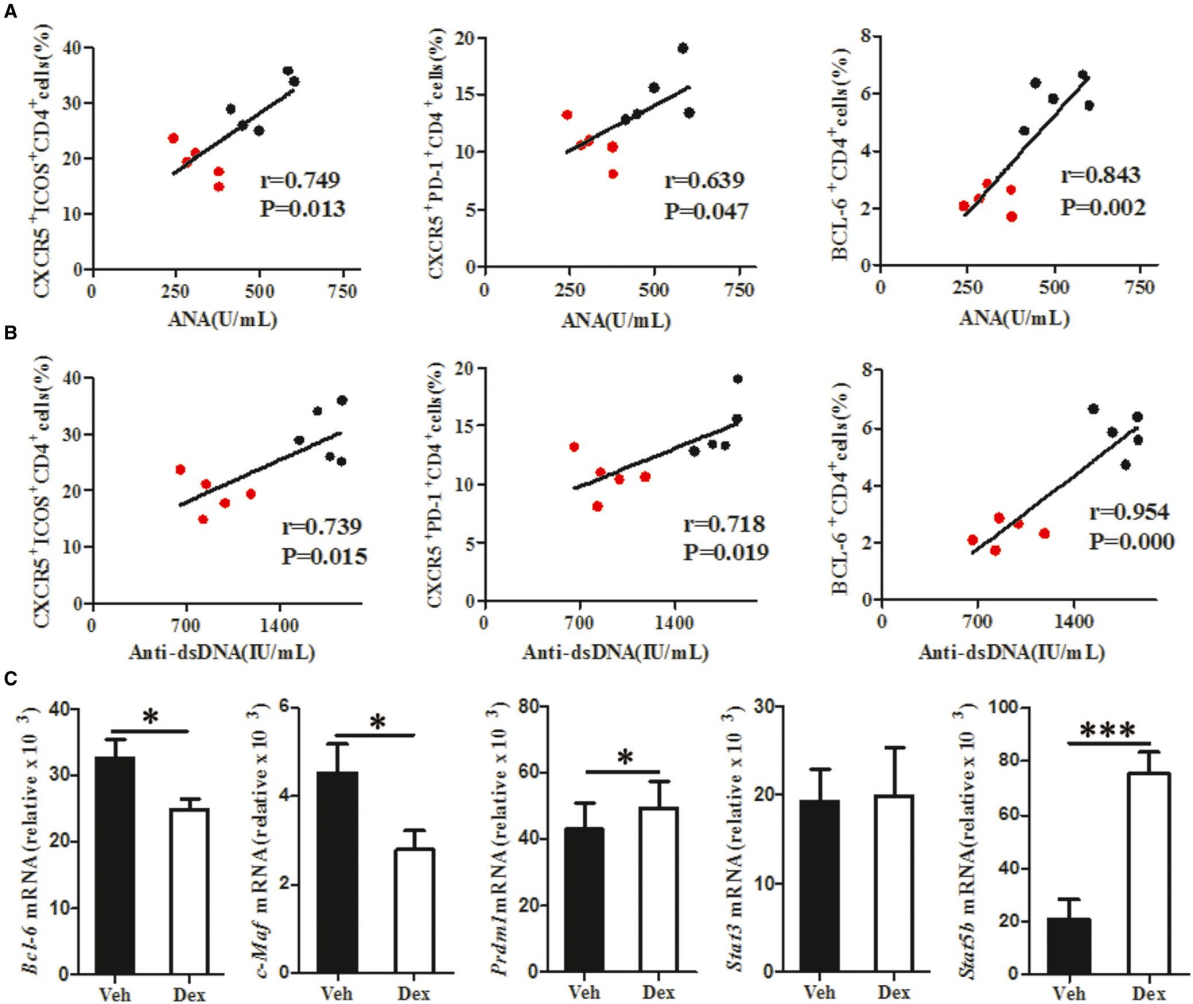
Potential mechanism underlying Dex effect on Tfh cell responses. Sixteen‐week‐old female MRL/lpr mice (36 ± 2 g) were treated with vehicle (normal saline; noted Veh) or 1 mg/kg of Dex (noted Dex) for 4 weeks. A Positive correlation between the percentages of Tfh cells (CD4^+^CXCR5^+^ICOS^+^, CD4^+^CXCR5^+^PD‐1^+^, or CD4^+^BCL‐6^+^) and the levels of serum anti‐dsDNA antibodies in MRL/lpr mice. B Positive correlation between the percentages of Tfh cells (CD4^+^CXCR5^+^ICOS^+^, CD4^+^CXCR5^+^PD‐1^+^, or CD4^+^BCL‐6^+^) and the levels of serum ANA in MRL/lpr. C Relative expression of transcription factor mRNAs in CD4^+^ T cells from spleen of MRL/lpr mice. *Bcl‐6, c‐Maf, Stat3, Prdm1* and *Stat5b* mRNA levels quantified by TaqMan PCR. Values are the mean and SD of 5 mice per group, (black represents Veh, red represents Dex ),*P < .05, **P < .01, and *** *P* < .001

Lupus is characterized by the overproduction of autoantibodies. To confirm the likely mechanism whereby Dex suppresses disease progression, we analysed the correlation between the frequency of Tfh cells, identified as CD4^+^CXCR5^+^ICOS^+^, CD4^+^CXCR5^+^PD‐1^+^ or CD4^+^BCL‐6^+^ cells, and serum ANA and anti‐dsDNA antibody levels. This analysis revealed a strong correlation between these biological parameters. These data indicate that Dex reduces ANA and anti‐dsDNA antibody levels through the inhibition of Tfh cell responses (Figure 5A‐B).

To explore the potential mechanism underlying the biological activity of Dex, we further assayed the expression of several transcription factors. We were surprised to find a significant down‐regulation of *Bcl‐6* and *c‐Maf* mRNA levels in the CD4^+^ T cells from MRL/lpr mice on treatment with Dex, paralleling the decrease in Tfh cell numbers. Meanwhile, *Prdm1* and *Stat5b* mRNA levels were up‐regulated in Dex‐treated MRL/lpr mice were up‐regulated. In contrast, there was no effect on the mRNA expression of *Stat3* (Figure 5C).

## 4 DISCUSION

SLE is an autoimmune disease that is characterized by the production of autoantibodies and multiorgan damage, especially nephritis.^30^, ^31^, ^32^ Previous studies have shown that Dex is able to reduce ANA and anti‐dsDNA antibody levels in MRL/lpr mice.^29^ In keeping with these studies, our data show that the levels of serum ANA and anti‐dsDNA antibodies were significantly decreased in Dex‐treated MRL/lpr mice. Interestingly, we also found that disease severity, splenomegaly, significant hyperproliferation and IgG deposits in kidney glomeruli were reduced on Dex treatment of MRL/lpr mice.

Previous studies have revealed that abnormal B‐cell activation and dysregulated GCs in secondary lymphoid tissues may play a crucial role in SLE pathology.^33^, ^34^ More recent work presented clear evidence that Tfh cells are indeed expanded in a subgroup of patients suffering from severe SLE.^19^, ^35^, ^36^ Tfh cells represent a novel subpopulation of CD4^+^T cells that provide indispensable help to B cells, particularly during the GC reaction.^36^, ^37^ Therefore, our present study reveals that Dex potentially exerts its therapeutic effects of Dex via inhibition of Tfh cell responses in lupus‐prone MRL/lpr mice.

Tfh cells are characterized by the surface molecules ICOS and PD‐1, and the transcription factor BCL‐6, which are also critical in B‐cell responses.^38^, ^39^, ^40^, ^41^ In our study in mouse, we found that the levels of ICOS, PD‐1 and BCL‐6 on Tfh cells were obviously decreased after treatment with Dex both in Balb/c and MRL/lpr mice. The well‐known signature cytokine of follicular helper T cells IL‐21 is multifunctional and affects the activation, differentiation and expansion of GC B cells. As such, IL‐21 is an essential participant in SLE pathogenesis.^42^, ^43^ Our results showed a decrease in the protein and mRNA levels of IL‐21 on Dex treatment in both Balb/c and MRL/lpr mice. BCL‐6 plays a critical role in regulating Tfh cells and is suppressed by Blimp‐1, the protein encoded by the gene *Prdm1*. The antagonizing interaction of *Bcl‐6* with *Prdm1* is essential for T‐cell differentiation.^22^, ^44^, ^45^, ^46^ Our data demonstrate that treatment of Balb/c or MRL/lpr mice with Dex reduces the expression of *Bcl‐6*, but increases the expression of *Prdm1*. Besides *Bcl‐6*, other transcription factors have also been described as mediators of Tfh cell differentiation. Among the positive inducers of Tfh cell differentiation is *c‐Maf*.^47^, ^48^ On the contrary, *Stat5* efficiently suppresses Tfh differentiation by reducing the transcription of Tfh‐associated genes, such as c‐Maf, *Bcl‐6* and *Il‐21*.^22^ ^,^ ^49‐52^ Our results show that *c‐Maf* and *Stat5* could also be regulated by Dex treatment. Thus, Dex most probably suppresses Tfh cell differentiation by affecting these transcription factors.

Tfh cells have a direct effect on B cells and facilitate GC formation.^13^, ^22^ Aberrant formation of GCs and abnormal number of GCs in B cells, which influence the production of autoantibodies, are also connected with the progression of SLE.^13^, ^14^, ^15^ Our findings show that the effects of Dex effect on Tfh cells result in an obvious decrease in the frequency of GCs in B cells in mice. Previous studies revealed that MRL/lpr mice display significantly higher levels of serum IgA, IgG and IgG subtypes. We were surprised to find that the levels of serum IgG, IgG2a/b and IgA were decreased significantly in Dex‐treated MRL/lpr mice.

Previous studies showed that the expansion of Tfh cells is closely associated with excessive production of anti‐dsDNA antibodies and severe end organ damage, such as nephritis.^18^, ^20^, ^21^ Our data showed a positive correlation between the frequency of Tfh cells and serum ANA and anti‐dsDNA antibody levels.

In conclusion, we demonstrated that the levels of autoantibodies in MRL/lpr mice were reduced when Tfh cell differentiation was inhibited by Dex treatment. Few studies have addressed the effects of Dex on Tfh cells. Therefore, our study sheds new light on a previously undescribed negative effect of Dex on Tfh cell differentiation and IL‐21 secretion, which are both up‐regulated in SLE patients compared to healthy controls. Our next step will be to investigate the effects of Dex on other cell populations, including CD4^+^T‐cell subpopulations (effector memory, central memory, naïve, and if possible regulatory T cells) and CD8^+^ T cells, in both MRL/lpr mice and SLE patients. We will also compare the combined effects of Dex with these populations and on Tfh cells with that of other glucocorticoids, on these populations and on Tfh cells.

## AUTHOR CONTRIBUTIONS

**Chunxiu Shen:** Conceptualization (equal); Data curation (lead); Formal analysis (lead); Methodology (equal); Writing‐original draft (lead); Writing‐review & editing (lead). **Xiaonan Xue:** Data curation (equal); Methodology (equal). **Xiaoyu Zhang:** Data curation (equal); Methodology (equal). **Lihua Wu:** Funding acquisition (supporting); Methodology (supporting). **xiangguo duan:** Conceptualization (lead); Project administration (lead). **Chunxia Su:** Conceptualization (lead); Data curation (lead); Formal analysis (lead); Funding acquisition (lead); Investigation (lead); Methodology (lead); Project administration (lead).

## ETHICAL APPROVAL

The Ningxia Medical University Ethics Review Committee approved this study.

## CONFLICT OF INTEREST

The authors report no declarations of interest.

## Supporting information

Fig S1

Fig S2

## References

[1] Nomura A, Noto D, Murayama G, Chiba A, Miyake S. Unique primed status of microglia under the systemic autoimmune condition of lupus‐prone mice. Arthritis Res Ther. 2019;21(1):303. 10.1186/s13075-019-2067-8 31888754 PMC6936062

[2] Abuaf N, Desgruelles C, Moumaris M, et al. Detection by flow cytometry of anti‐DNA autoantibodies and circulating DNA immune complexes in lupus erythematosus. J Immunol Res. 2019;2019:1–12. 10.1155/2019/6047085 PMC6915121 31886305

[3] Yuan F, Harder J, Ma J, Yin X, Zhang X, Kosiewicz MM. Using multiple analytical platforms to investigate the androgen depletion effects on fecal metabolites in a mouse model of systemic lupus erythematosus. J Proteome Res. 2020;19(2):667–676. 10.1021/acs.jproteome.9b00558 31820642 PMC7123809

[4] Ma K, Du W, Wang X, et al. Multiple functions of B cells in the pathogenesis of systemic lupus erythematosus. Int J Mol Sci. 2019;20(23):6021. 10.3390/ijms20236021 PMC6929160 31795353

[5] Breitfeld D, Ohl L, Kremmer E, et al. Follicular B helper T cells express CXC chemokine receptor 5, localize to B cell follicles, and support immunoglobulin production. J Exp Med. 2000;192:1545–1552. 10.1084/jem.192.11.1545 11104797 PMC2193094

[6] Akiba H, Takeda K, Kojima Y, et al. The role of ICOS in the CXCR5+ follicular B helper T cell maintenance in vivo. J Immunol. 2005;175:2340–2348. 10.4049/jimmunol.175.4.2340 16081804

[7] Good‐Jacobson KL, Szumilas CG, Chen L, Sharpe AH, Tomayko MM, Shlomchik MJ. PD‐1 regulates germinal center B cell survival and the formation and affinity of long‐lived plasma cells. Nat Immunol. 2010;11:535–542. 10.1038/ni.1877 20453843 PMC2874069

[8] Bauquet AT, Jin H, Paterson AM, et al. The costimulatory molecule ICOS regulates the expression of c‐Maf and IL‐21 in the development of follicular T helper cells and TH‐17 cells. Nat Immunol. 2009;10:167–175. 10.1038/ni.1690 19098919 PMC2742982

[9] Crotty S. Follicular helper CD4 T cells (TFH). Annu Rev Immunol. 2011;29:621–663. 10.1146/annurev-immunol-031210-101400 21314428

[10] Park HJ, Kim DH, Lim SH, et al. Insights into the role of follicular helper T cells in autoimmunity. Immune Netw. 2014;14:21–29. 10.4110/in.2014.14.1.21 24605077 PMC3942504

[11] Liu X, Yan X, Zhong B, et al. Bcl6 expression specifies the T follicular helper cell program in vivo. J Exp Med. 2012;209(10):1841–1852. 10.1084/jem.20120219 22987803 PMC3457730

[12] Crotty S, Johnston RJ, Schoenberger SP. Effectors and memories: Bcl‐6 and Blimp‐1 in T and B lymphocyte differentiation. Nat Immunol. 2010;11:114–120. 10.1038/ni.1837 20084069 PMC2864556

[13] Duan X, Sun P, Lan Y, et al. 1IFN‐alpha modulates memory Tfh cells and memory B cells in mice, following recombinant FMDV adenoviral challenge. Front Immunol. 2020;11:701. 10.3389/fimmu.2020.00701 32411135 PMC7200983

[14] Su C, Duan X, Zheng J, Liang L, Wang F, Guo L. IFN‐α as an adjuvant for adenovirus‐vectored FMDV subunit vaccine through improving the generation of T follicular helper cells. PLoS One. 2013;8(6):e66134. 10.1371/journal.pone.0066134 23823532 PMC3688841

[15] Blanco P, Ueno H, Schmitt N. T follicular helper (Tfh) cells in lupus: activation and involvement in SLE pathogenesis. Eur J Immunol. 2016;46:281–290. 10.1002/eji.201545760 26614103

[16] Vinuesa CG, Cook MC, Angelucci C, et al. A RING‐type ubiquitin ligase family member required to repress follicular helper T cells and autoimmunity. Nature. 2005;435(7041):452–458. 10.1038/nature03555 15917799

[17] Odegard JM, Marks BR, DiPlacido Leah D, et al. ICOSdependent extrafollicular helper T cells elicit IgG production via IL‐21 in systemic autoimmunity. J Exp Med. 2008;205(12):2873–2886. 10.1084/jem.20080840 18981236 PMC2585848

[18] Jang SG, Lee J, Hong S‐M, et al. Niclosamide suppresses the expansion of follicular helper T cells and alleviates disease severity in two murine models of lupus via STAT3. J Transl Med. 2021;19(1):86. 10.1186/s12967-021-02760-2 33632240 PMC7908700

[19] Linterman MA, Rigby RJ, Wong RK, et al. Follicular helper T cells are required for systemic autoimmunity. J Exp Med. 2009;206(3):561–576. 10.1084/jem.20081886 19221396 PMC2699132

[20] Feng X, Wang D, Chen J, et al. Inhibition of aberrant circulating Tfh cell proportions by corticosteroids in patients with systemic lupus erythematosus. PLoS One. 2012;7:e51982. 10.1371/journal.pone.0051982 23284839 PMC3524129

[21] Simpson N, Gatenby PA, Wilson A, et al. Expansion of circulating T cells resembling follicular helper T cells is a fixed phenotype that identifies a subset of severe systemic lupus erythematosus. Arthritis Rheum. 2010;62:234–244. 10.1002/art.25032 20039395

[22] Duan X, Shen C, Zhang X, et al. Toll‐like receptor 7 agonist imiquimod prevents the progression of SLE in MRL/lpr mice via inhibiting the differentiation of T follicular helper cells. Int Immunopharmacol. 2020;80:106239. 10.1016/j.intimp.2020.106239 32007709

[23] Simpson N, Gatenby PA, Wilson A, et al. Expansion of circulating T cells resembling follicular helper T cells is a fixed phenotype that identifies a subset of severe systemic lupus erythematosus. Arthritis Rheum. 2010;62(1):234–244. 10.1002/art.25032 20039395

[24] Du J, Li M, Zhang D, et al. Flow cytometry analysis of glucocorticoid receptor expression and binding in steroid‐sensitive and steroid‐resistant patients with systemic lupus erythematosus. Arthritis Res Ther. 2009;11:R108. 10.1186/ar2763 19594946 PMC2745790

[25] Ho CY, Wong CK, Li EK, Lam WK. Effects of dexamethasone on the expression of Fas molecules and apoptosis of lymphocytes in patients with systemic lupus erythematosus. Immunol Invest. 2001;30:231–243. 10.1081/imm-100105 11570643

[26] Huaxia Y, Huazhen L, Ziyue Z, et al. Management of severe refractory systemic lupus erythematosus: real‐world experience and literature review. Clin Rev Allergy Immunol. 2021;60(1):17–30. 10.1007/s12016-020-08817-2 33159635

[27] Giles AJ, Hutchinson M‐KND, Sonnemann HM, et al. Dexamethasone‐induced immunosuppression: mechanisms and implications for immunotherapy. J Immunother Cancer. 2018;6(1):51. 10.1186/s40425-018-0371-5 29891009 PMC5996496

[28] Yanwei W, Shijun H, Bingxin B, et al. Therapeutic effects of the artemisinin analog SM934 on lupus‐prone MRL/lpr mice via inhibition of TLR‐triggered B‐cell activation and plasma cell formation. Cell Mol Immunol. 2016;13(3):379–390. 10.1038/cmi.2015.13 25942599 PMC4856803

[29] Yang C, Xue J, An N, et al. Accelerated Glomerular cell senescence in experimental lupus nephritis. Med Sci Monit. 2018;24:6882–6891. 10.12659/msm.909353 30265659 PMC6180956

[30] Lou H, Wojciak‐Stothard B, Ruseva MM, et al. Autoantibody‐dependent amplification of inflammation in SLE. Cell Death Dis. 2020;11(9):729. 10.1038/s41419-020-02928-6 32908129 PMC7481301

[31] Melki I, Allaeys I, Tessandier N, et al. FcγRIIA expression accelerates nephritis and increases platelet activation in systemic lupus erythematosus. Blood. 2020;136(25):2933–2945. 10.1182/blood.2020004974 33331924 PMC7751357

[32] Yang X, Yang J, Chu Y, et al. T follicular helper cells mediate expansion of regulatory B cells via IL‐21 in lupus‐prone MRL/lpr mice. PLoS One. 2013;8:e62855. 10.1371/journal.pone.0062855 23638156 PMC3634758

[33] Patrick S, Katharina W, Lang AB, Martin L, Pius L, Bernhard M. Cxc chemokine receptor 5 expression defines follicular homing T cells with B cell helper function. J Exp Med. 2000;192(11):1553–1562. 10.1084/jem.192.11.1553 11104798 PMC2193097

[34] Kurata I, Matsumoto I, Sumida T. T follicular helper cell subsets: a potential key player in autoimmunity. Immunol Med. 2020;44(1):1–9. 10.1080/25785826.2020.1776079 32546108

[35] Di Y, Vinuesa CG. The elusive identity of T follicular helper cells. Trends Immunol. 2010;31(10):377–383. 10.1016/j.it.2010.07.001 20810318

[36] Dong L, He Y, Cao Y, et al. Functional differentiation and regulation of follicular T helper cells in inflammation and autoimmunity. Immunology. 2021;163(1):19–32. 10.1111/imm.13282 33128768 PMC8044332

[37] Song W, Craft J. T follicular helper cell heterogeneity: time, space, and function. Immunol Rev. 2019;288:85–96. 10.1111/imr.12740 30874350 PMC6422039

[38] Qi H. T follicular helper cells in space‐time. Nat Rev Immunol. 2016;16:612–625. 10.1038/nri.2016.94 27573485

[39] Victora GD, Schwickert TA, Fooksman DR, et al. Germinal center dynamics revealed by multiphoton microscopy with a photoactivatable fluorescent reporter. Cell. 2010;143(4):592–605. 10.1016/j.cell.2010.10.032 21074050 PMC3035939

[40] Tarlinton D, Good‐Jacobson K. Diversity among memory B cells: origin, consequences, and utility. Science. 2013;341:1205–1211. 10.1126/science.1241146 24031013

[41] Long‐Shan J, Xue‐Hua S, Xin Z, et al. Mechanism of follicular helper T cell differentiation regulated by transcription factors. J Immunol Res. 2020;2020:1–9. 10.1155/2020/1826587 PMC7387970 32766317

[42] Zou X, Wang S, Zhang Y, Wang X, Yang W. The role of follicular T helper cells in the onset and treatment of type 1 diabetes. Int Immunopharmacol. 2020;84:106499. 10.1016/j.intimp.2020.106499 32298964

[43] Johnston RJ, Poholek AC, DiToro D, et al. Bcl6 and Blimp‐1 are reciprocal and antagonistic regulators of T follicular helper cell differentiation. Science. 2009;325(5943):1006–1010. 10.1126/science.1175870 19608860 PMC2766560

[44] Diehl SA, Schmidlin H, Nagasawa M, et al. STAT3‐mediated up‐regulation of BLIMP1 is coordinated with BCL6 down‐regulation to control human plasma cell differentiation. J Immunol. 2008;180(7):4805–4815. 10.4049/jimmunol.180.7.4805 18354204 PMC2396731

[45] Crotty S. T follicular helper cell differentiation, function, and roles in disease. Immunity. 2014;41:529–542. 10.1016/j.immuni.2014.10.004 25367570 PMC4223692

[46] Liu X, Nurieva RI, Dong C. Transcriptional regulation of follicular T‐helper (Tfh) cells. Immunol Rev. 2013;252:139–145. 10.1111/imr.12040 23405901 PMC3579502

[47] Johnston RJ, Soo CY, Diamond JA, Yang JA, Shane C. STAT5 is a potent negative regulator of TFH cell differentiation. J Exp Med. 2012;209(2):243–250. 10.1084/jem.20111174 22271576 PMC3281266

[48] Ma CS, Deenick EK, Marcel B, Tangye SG. The origins, function, and regulation of T follicular helper cells. J Exp Med. 2012;209(7):1241–1253. 10.1084/jem.20120994 22753927 PMC3405510

[49] Ryu H, Kim J, Kim D, Lee JE, Chung Y. Cellular and molecular links between autoimmunity and lipid metabolism. Mol Cells. 2019;42(11):747–754. 10.14348/molcells.2019.0196 31766832 PMC6883973

