# Corrigendum

**DOI:** 10.1111/jcmm.17123

**Published:** 2022-01-08

In Chunxiu Shen et al.,^1^ the HE‐stained picture of MRL/lpr + Dex group in Figure 1c is incorrect. The correct figure is shown below. The authors confirm all results and conclusions of this article remain unchanged.

**FIGURE 1:**
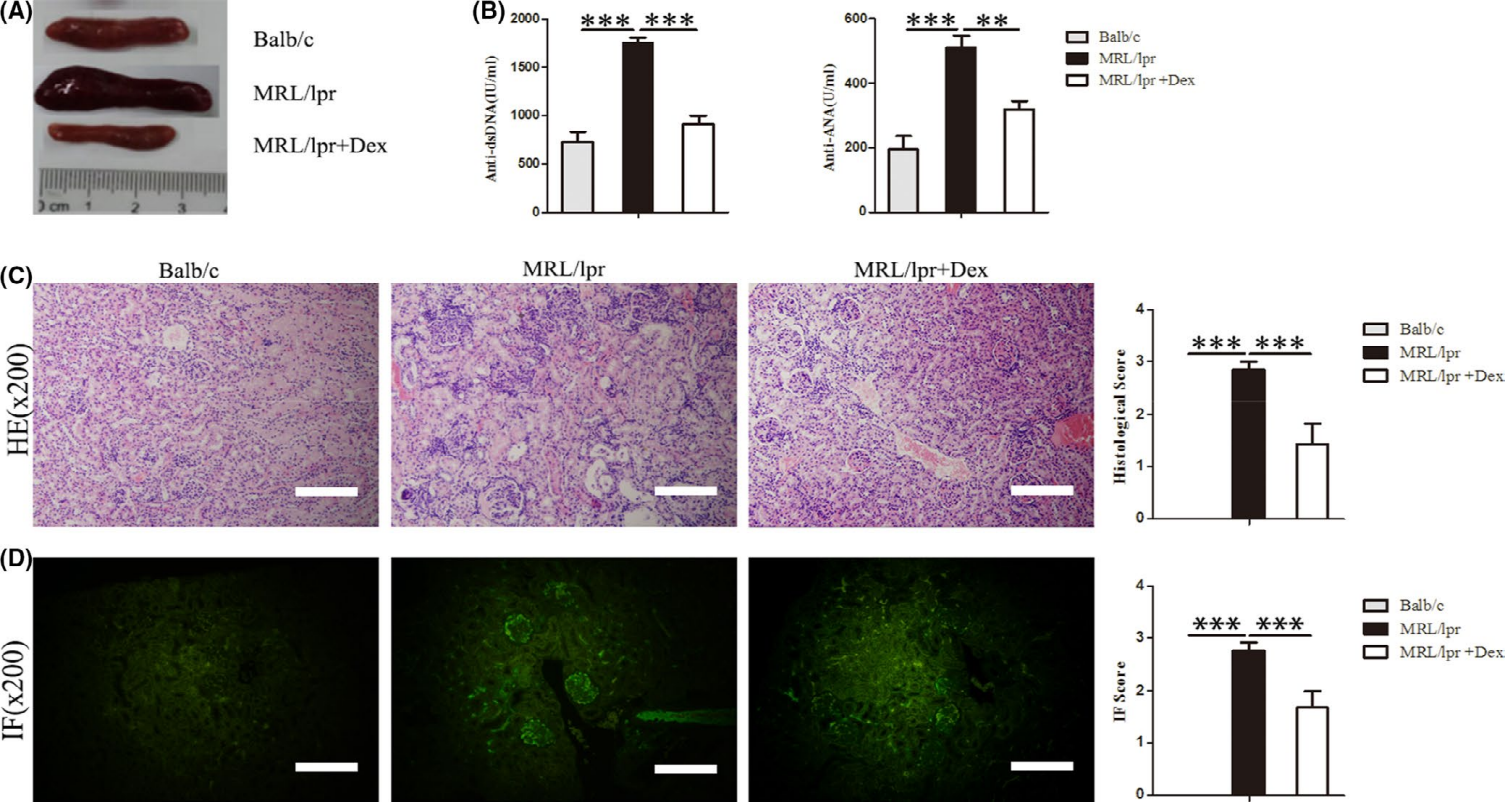
Effects of Dex on the lupus syndromes of MRL/lpr mice. Sixteen‐week‐old female MRL/lpr mice (36 ± 2 g) were treated with vehicle (normal saline) or 1 mg/kg of Dex for 4 weeks, age‐matched Balb/c mice as the normal control group. (A) Splenomegaly in MRL/lpr mice and alleviated after Dex treatment. (B) The serum levels of anti‐dsDNA antibodies and ANA. (C) Sections of kidney tissue were stained with H&E and semi‐quantitative analysis of the histological score. (D) Sections of kidney tissue were stained with immunofluorescence IgG and semi‐quantitative analysis of glomerular IgG deposition. Original magnification ×200. The scale bar in each image represents 100 μm. Values are the mean and SD of 5 mice per group, **p < .01 and ***p < .001
