# Supplementary figures and images for: Dexamethasone reduces autoantibody levels in MRL/lpr mice by inhibiting Tfh cell responses
Source: J Cell Mol Med. 2021 Jul 28;25(17):8329–37. doi: 10.1111/jcmm.16785 (PMC8419171; doi:10.1111/jcmm.16785)

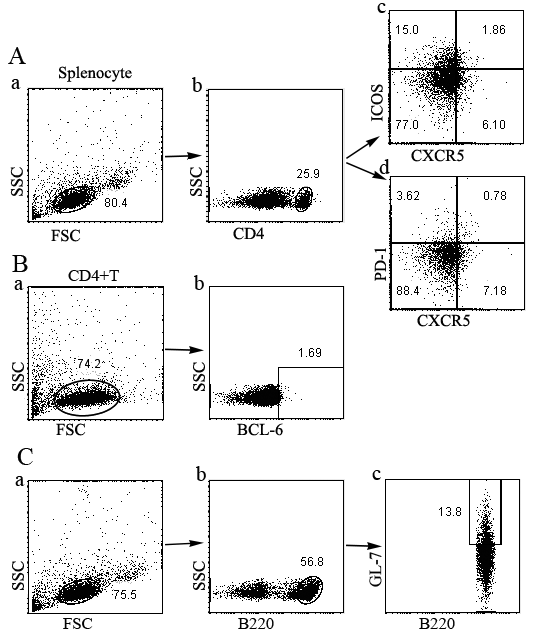

Supplement: Supplementary file 1 — Fig S1 [file JCMM-25-8329-s002.tif]

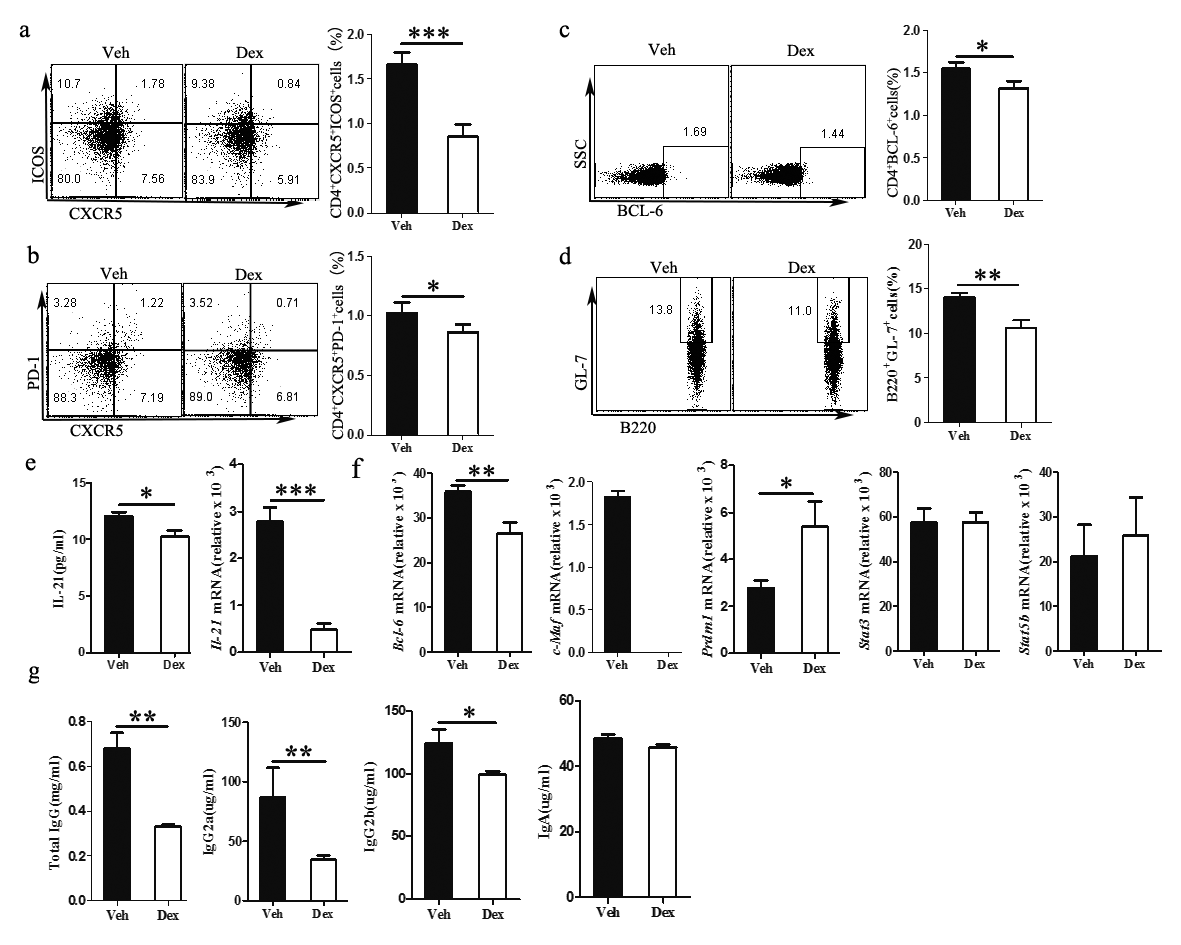

Supplement: Supplementary file 2 — Fig S2 [file JCMM-25-8329-s001.tif]
